# Increased Purinergic Responses Dependent on P2Y2 Receptors in Hepatocytes from CCl_4_-Treated Fibrotic Mice

**DOI:** 10.3390/ijms21072305

**Published:** 2020-03-26

**Authors:** Erandi Velázquez-Miranda, Christian Molina-Aguilar, Adriana González-Gallardo, Olivia Vázquez-Martínez, Mauricio Díaz-Muñoz, Francisco G Vázquez-Cuevas

**Affiliations:** Departamento de Neurobiología Celular y Molecular, Instituto de Neurobiología, Universidad Nacional Autónoma de México, Querétaro 76230, Mexico; erandivelazquez@gmail.com (E.V.-M.); cmolina@liigh.unam.mx (C.M.-A.); gallardog@unam.mx (A.G.-G.); ovazquez@comunidad.unam.mx (O.V.-M.); mdiaz@comunidad.unam.mx (M.D.-M.)

**Keywords:** purinergic signaling, P2Y2 receptor, adenine nucleotides, hepatocyte, CCl_4_, liver fibrosis

## Abstract

Inflammatory and wound healing responses take place during liver damage, primarily in the parenchymal tissue. It is known that cellular injury elicits an activation of the purinergic signaling, mainly by the P2X7 receptor; however, the role of P2Y receptors in the onset of liver pathology such as fibrosis has not been explored. Hence, we used mice treated with the hepatotoxin CCl_4_ to implement a reversible model of liver fibrosis to evaluate the expression and function of the P2Y2 receptor (P2Y2R). Fibrotic livers showed an enhanced expression of P2Y2R that eliminated its zonal distribution. Hepatocytes from CCl_4_-treated mice showed an exacerbated ERK-phosphorylated response to the P2Y2R-specific agonist, UTP. Cell proliferation was also enhanced in the fibrotic livers. Hepatic transcriptional analysis by microarrays, upon CCl_4_ administration, showed that P2Y2 activation regulated diverse pathways, revealing complex action mechanisms. In conclusion, our data indicate that P2Y2R activation is involved in the onset of the fibrotic damage associated with the reversible phase of the hepatic damage promoted by CCl_4_.

## 1. Introduction

Extracellular nucleotides, as intercellular messengers, exert their actions through specific receptors known as P2X and P2Y. While P2X receptors are ligand-gated ion channels, P2Y receptors (P2YRs) belong to the superfamily of G-protein coupled receptors (GPCRs) with seven transmembrane segments. Eight P2YRs have been described to date (P2Y1, P2Y2, P2Y4, P2Y6, P2Y11-14). P2Y2R is a prototypical member of the family that displays its actions through Gαq coupling and the phosphoinositide-Ca^2+^ pathway [1].

The expression of P2Y2R has been described in liver cells. In primary cultures of human hepatocytes, P2Y2R activity induced an increment in the levels of intracellular calcium [Ca^2+^]_i_, inositol polyphosphates, glycogen phosphorylase activity and MAPK-ERK phosphorylation [2]. In rat hepatocytes, stimulation with a non-hydrolysable analog of ATP, ATPγS, induced cell proliferation and an increment in the phosphorylation of JNK. ATPγS also induced the early response genes c-fos and c-jun and the binding of the AP-1 complex to DNA, suggesting activation of this effector [3].

Importantly, it has been demonstrated that P2Y2R is necessary for liver growth after partial hepatectomy. In P2Y2R^-/-^ mice, proliferative signals like ERK phosphorylation, egr-1 activation and AP-1 complex binding to DNA, which are responsible for priming the regenerative process after 70% resection of the liver, are impaired [4].

In the context of tissue damage, ATP released from injured tissues is considered a damage associated molecular pattern (DAMP) [5,6]. It is well understood that extracellular ATP, acting mainly through P2X7R, induces the innate inflammatory response by promoting the activity of the inflammasome NLRP3 [7], which has been shown to be particularly relevant during liver fibrosis [8]. However, it remains unknown whether P2YRs play a role in response to cellular damage. In the fibrotic context, the signaling associated with P2Y2R has not been characterized. Since fibrosis is an aberrant wound healing process where hepatocyte proliferation has lost control, we reasoned that extracellular ATP released through chronic liver injury has a role in fibrotic development, thus influencing cell proliferation.

Recently, it was demonstrated that P2Y2R plays a relevant role in tumor initiation in hepatocarcinogenesis, since P2Y2^-/-^ mice develop a lower number of liver tumors in response to diethylnitrosamine (DEN) injection. Mechanistically, this effect was correlated with a decreased cell proliferation in the early stage of the response to the hepatotoxic [8].

Therefore, it is possible to hypothesize that extracellular purines play complex roles in liver fibrosis that involve various receptor subtypes, with P2X7 mediating inflammation and P2Y2 promoting proliferation.

Although the fibrosis-induced hepatocellular damage involves an interplay among multiple liver cell types, parenchymal cells are the primary sensors of injury signals and are primed for pro-fibrotic responses [5,9]; under this rationale, we are interested in the responses mediated by P2Y2R in hepatocytes.

In the present study, we analyzed the expression and function of P2Y2R in a mouse model of fibrosis, induced by the administration of CCl_4_ for 4 weeks. P2Y2R was up-regulated, and the UTP-induced purinergic responses, detected as ERK phosphorylation and cell proliferation, were exacerbated. The gene expression landscape indicated that extracellular purines through P2Y2R influenced a complex web of informational pathways to support the survival of fibrotic hepatocytes. We propose that P2Y2R is an important element in the hepatic fibrotic changes, mainly driving the parenchymal proliferative responses associated with fibrosis installation.

## 2. Results

### 2.1. Hepatic Fibrosis Induced by CCl_4_

**Figure 1:**
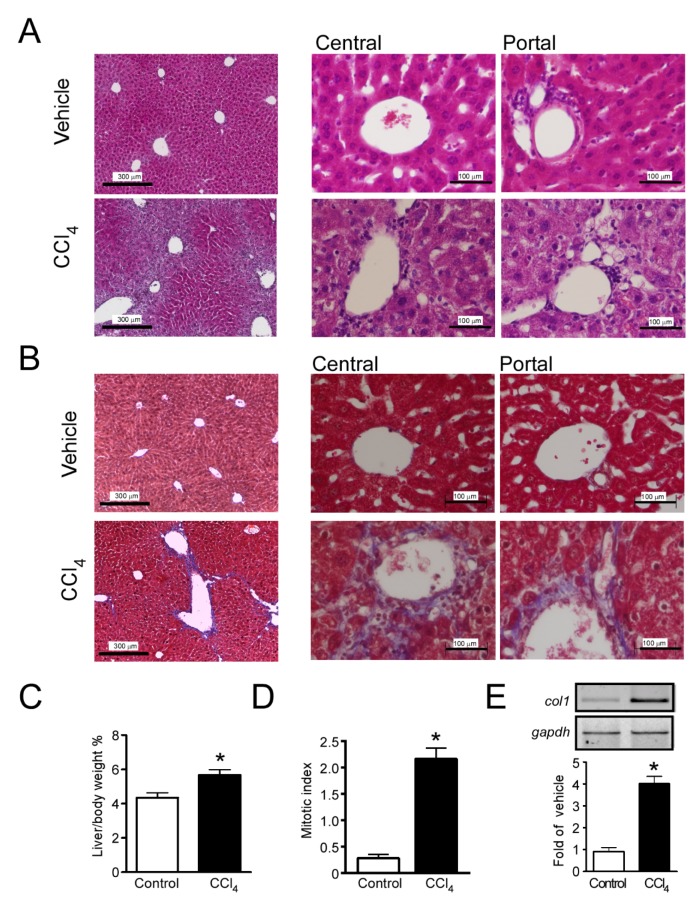
Administration of CCl_4_ for 4 weeks induced a fibrotic phenotype. Mice of the C57BL/6 strain were treated with intraperitoneal injections of CCl_4_, three times a week for 4 weeks, then, a histological analysis of liver sections was performed. Hematoxylin and Eosin (**A**) and Masson’s Trichrome stain (**B**) in 5 µm liver slices, showing low magnification fields and central and portal regions, from control and CCl_4_-injected mice (*n*= three mice, three slides each). (**C**) Percent of liver/body weight from control- or CCl_4_-injected mice. (**D**) Effects of CCl_4_ treatment on the rate of liver mitosis (mitotic index) and E) Expression level of *Col1a1* transcript in liver homogenates as a marker of fibrosis. * *p* < 0.05, Student’s *t-*test. For C, data are the mean of three mice. In (**D**), results are the average of 72 fields from three livers of each group and in (**E**), five mice per group were analyzed.

The use of CCl_4_ to induce liver fibrosis is a well-characterized model [10,11]; in the present study, we induced a reversible fibrotic state, as described in the Material and Methods section. Histopathological assessment by hematoxylin-and eosin-staining in the livers of control mice (Figure 1A) showed healthy hepatocytes with granular eosinophil cytoplasm and intact nuclei with clearly defined nucleoli. Tissue architecture showed normal portal tracts with portal venules, hepatic arterioles and interlobular bile ducts without disturbances and a well-preserved sinusoidal space between hepatic cords (Figure 1A). Using Masson’s trichrome staining, thin collagen fibers were observed only within the pericentral zones (Figure 1B).

In mice treated for 4 weeks with CCl_4_, tissue and cytological abnormalities were evident. Portal and central zones presented extensive areas with anisocytosis (evidence of the inflammatory state). Hepatocytes exhibited fat vacuoles and a granular eosinophilic cytoplasm, as well as a high incidence of dismembered nuclei, pyknosis and karyolysis. This group also presented signs of necrosis and inflammation, such as a high lymphocyte count in the portal and central zones, and loss of Disse space (Figure 1A). Using Masson´s trichrome staining, we observed coarse collagen fibers in the portal zone with ramifications for the central zone. The tissue also showed hyperplasia and the neo-formation of bile ducts as a response to the toxic damage (Figure 1B). Moreover, mice treated with CCl_4_ showed hepatomegaly (4.3 ± 0.3 vs. 5.7% ± 0.4% liver/body weight for control and CCl_4_, respectively, *p* = 0.0021, Student’s *t-*test, *n* = 4) (Figure 1C), a characteristic of the induction of the fibrotic state.

To confirm the pro-fibrotic state of the livers in the CCl_4_-treated mice, the mitotic index was analyzed. We observed that livers from CCl_4_-treated mice showed an increased frequency of mitotic hepatocytes (0.30 ± 0.07 vs. 2.2 ± 0.2 mitosis by field, *p* < 0.0001, Student’s *t*-test) (Figure 1D). Furthermore, induction of the gene coding for the α-1 chain of collagen type I in mice (C*ol1a1*) was analyzed by RT-PCR and compared to the constitutive transcript G*apdh*. A significant increment in the C*ol1a1* transcript was quantified when compared with control animals (4.0 ± 0.3 fold of control, *p* < 0.0001, Student’s *t*-test) (Figure 1E).

### 2.2. CCl_4_-Induced Changes in the Expression Level of P2Y Receptor Transcripts

**Figure 2:**
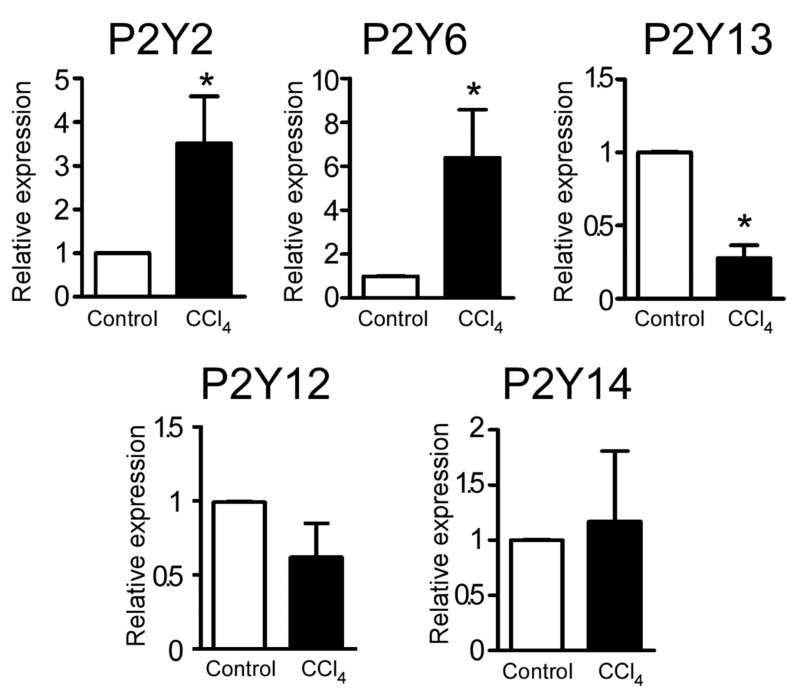
Effect of CCl_4_-administration on the expression level of P2YRs in mouse liver homogenates. Relative expression level of *P2yr2, P2yr6, P2yr12, P2yr13* and *P2yr14* from vehicle- and CCl_4_-injected mice analyzed by qPCR; data are expressed as fold of control group. * *p* < 0.05, Student’s *t-*test. *n* = 6 for *P2yr2* and *P2yr6*; and 5 for *P2yr12, P2yr13* and *P2yr14*.

Although this study focused on P2Y2R, we were interested in describing the changes in the expression levels of all P2YRs associated with the fibrotic state. To analyze if hepatocellular damage modified the expression levels of P2YRs, total RNA from liver homogenates of control and CCl_4_-treated mice was isolated and used for reverse transcription and qPCR. The transcript for *P2yr11* was not included in the analysis because this receptor is not expressed in rodents [12]. Interestingly, it was not possible to amplify *P2yr1* and *P2yr4* transcripts in both groups. An increment in the expression of *P2yr2* (6.3 ± 2.1 fold of control, *p* = 0.007, Student’s *t*-test) and *P2yr6* (3.1 ± 0.6 fold of control, *p* = 0.023, Student’s *t*-test) transcripts was noted, as well a reduction in the expression of *P2yr13* (0.20 ± 0.08 fold of control, *p* < 0.0001, Student’s *t*-test), whereas *P2yr14* and *P2yr12* did not show any changes (Figure 2).

Our main interest was the uridine nucleotide-sensitive P2YRs, particularly P2Y2R, because of its regulatory role in hepatic regeneration mediating proliferative pathways in hepatocytes [4]. Considering that liver fibrosis involves a deregulated wound-healing process, we decided to analyze further the role of P2Y2R in the reversible fibrotic process associated with CCl_4_-treatment.

### 2.3. The Fibrotic State Induced by CCl4 Alters the Distribution and Abundance of P2Y2R

**Figure 3:**
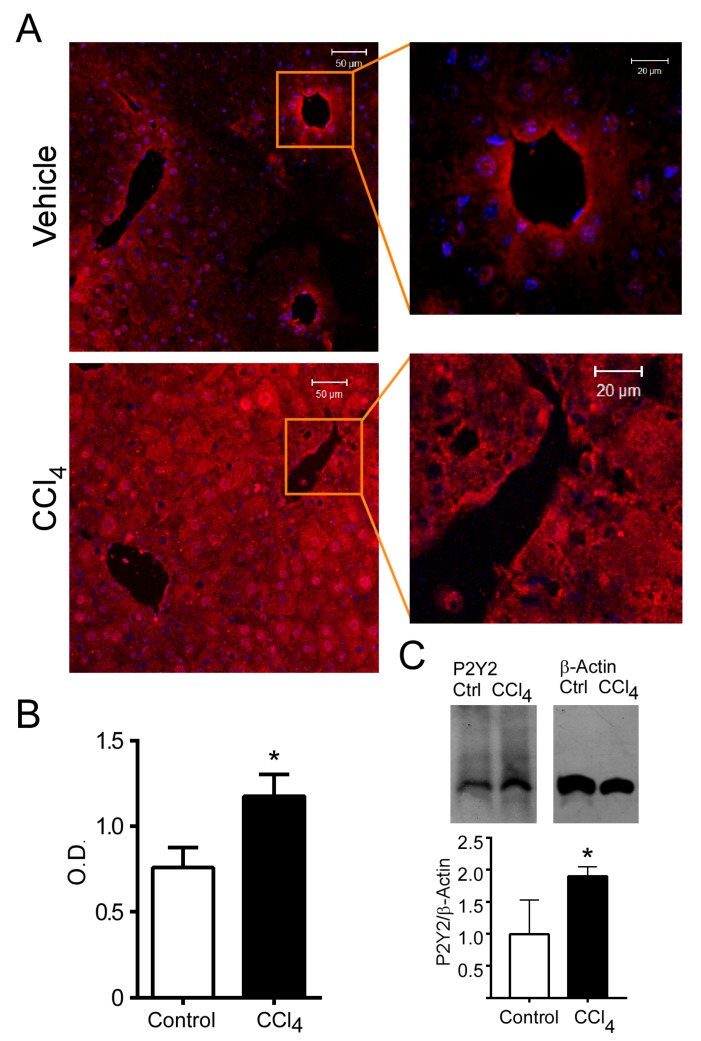
Detection of P2Y2R in liver sections from control and CCl_4_-treated mice. (**A**) Liver slices (5 µm) from vehicle- and CCl_4_-injected mice were labelled by immunofluorescence using a primary antibody directed against the carboxy end of the receptor and a secondary antibody coupled to Cy3 (red signal). (**B**) Total fluorescence was quantified, *n* = three mice, three slides each, in (**C**) the expression level of P2Y2R was analyzed in samples from control and CCl_4_-treated hepatocytes by Western blot and corrected against β-actin as constitutive protein, * *p* < 0.05 *Student’s t-test*. *n* = 3.

P2Y2R abundance and expression patterns were analyzed by immunofluorescence. In the livers of control mice, P2Y2R showed a clear zonal distribution. P2Y2R was expressed in the perivascular region of both central and portal zones. In contrast, CCl_4_ treatment elicited a general increment in the expression of P2Y2R in the entire liver (0.6 ± 0.1 vs. 1.8 ± 0.2 OD, for control and CCl_4_ groups respectively, *p* = 0.031 Student’s *t*-test*, n* = three mice, three slides each), resulting in the loss of the P2Y2R hepatic zonation (Figure 3A,B). The increment in the expression level of P2Y2R in CCl_4_-treated mice was confirmed by Western blotting (122 ± 24 vs. 179 ± 14 OD, control and CCl_4_ groups respectively, *p* = 0.036, Student’s *t*-test *n* = 3) (Figure 3C).

### 2.4. P2Y2R Function is Exacerbated in Hepatocytes from CCl_4_-Treated Mice

**Figure 4:**
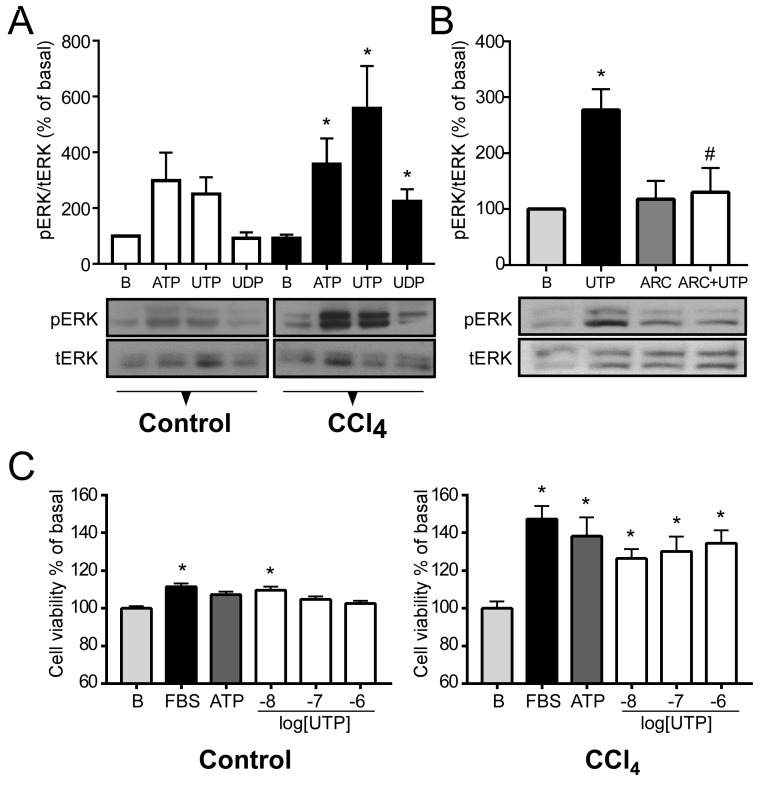
Analysis of P2Y2R function in primary cultured hepatocytes from control and fibrotic mice. (**A**) Induction of ERK phosphorylation by purinergic agonists in hepatocytes isolated from control and CCl_4_-treated mice (*n* = three controls and four treated). Hepatocytes were stimulated with 100 µM of ATP, UTP and UDP for 5 min. Level of phosphorylated ERK (pERK) was analyzed by Western blot; membranes were striped and re-blotted against total ERK (tERK); the ratio pERK/tERK is shown. (**B**) Primary cultures of CCl_4-_treated hepatocytes were stimulated for 5 min with 100 µM of UTP with and without the P2Y2R antagonist ARC118925 (ARC) 1 µM; the antagonist was preincubated for 20 min before the UTP addition (*n* = three cultures). (**C**) Effect of FBS, ATP and UTP on cell viability in hepatocytes isolated from control and CCl_4_-treated mice (*n* = five for each group). Hepatocytes were isolated and cultured for 16 h; afterwards, they were cultured for 4 h in FBS-free culture medium. Then, they were cultured with the indicated stimulus for 24 h. Cell viability was estimated by the tetrazolium salt method (MTS). Data were normalized to control cells (cultured in FBS-free medium). * *p* < 0.05 Student’s *t*-test vs. basal (**B**); # *p* < 0.05 Student’s *t*-test vs. UTP.

With the aim of verifying whether the changes observed in the expression levels of P2Y2R in fibrotic livers could have a functional impact, we decided to stimulate hepatocytes isolated from control or CCl_4_-treated mice with purinergic agonists and evaluate ERK phosphorylation and cell viability. In control hepatocytes, pharmacological stimulation with ATP, UTP and UDP, at a 100 µM concentration, showed a tendency to increment the phosphorylation level of ERK (298.4% ± 100.6%, 250.1% ± 59.9% and 90.7% ± 22.0 % of basal, respectively, ns); however, in hepatocytes from fibrotic mice, the same protocol promoted significant elevations in ERK phosphorylation (357.3% ±92.4% for ATP, *p* = 0.02; 557.3% ± 152.4% for UTP, *p* = 0.02 and 222.4% ± 44.9% for UDP, *p* = 0.03; Student’s *t-*test) (Figure 4A).

To support the specificity of the UTP stimulus over P2Y2 receptor, we used the selective P2Y2R antagonist ARC118925 [13]; thus, ARC abolished the ERK phosphorylation induced by UTP (277.1% ± 37.3% and 130.2% ± 43.3 % of basal, respectively, *p* = 0.04, Student’s *t*-test), showing that P2Y2R is mediating the UTP actions in primary cultures of mice hepatocytes (Figure 4B). The exclusive role of P2Y2R was supported by PCR analysis (Figure 2, Section 2.2) since no transcript of P*2ry4* was amplified in cDNAs of primary cultured hepatocytes, suggesting that P2Y4 is not expressed in this cell type.

In addition, we used the MTS assay to analyze the proliferative activity of primary hepatocyte cultures in response to different stimuli with the idea that mitochondrial activity would be directly related to cell proliferation. Although significantly different from basal levels (B) in both conditions, hepatocytes from CCl_4_-treated mice were more sensitive to a mitogenic stimulus (FBS) than vehicle-treated hepatocytes (147.4% ± 6.8% vs. 111.0% ± 1.6% of B respectively, *p* < 0.0001, Student’s *t*-test) (Figure 4C). Stimulation with ATP only induced a response from hepatocytes from fibrotic livers (138.2% ± 10.1% vs 107.0% ± 1.6% of B, for CCl_4_ and control groups respectively, *p* = 0.003, Student’s *t*-test). Hepatocytes from CCl_4_-treated animals were more sensitive to stimulation with UTP. In hepatocytes from CCl_4_-treated mice, 100 nM UTP elicited a response of 130.3% ± 7.6% *p* = 0.002 vs B, Student’s *t*-test; while in control hepatocytes the increment was only marginal, 106.5% ± 1.9%, ns. At 10 nM, values were of 126.5% ± 4.9%, (*p* = 0.001 vs B) and 111.1% ± 2.3% (*p* = 0.01 vs B, Student’s *t*-test) for CCl_4_-treated and control groups respectively, (Figure 4C). Our results suggest that CCl_4_ treatment sensitizes hepatocytes to purinergic stimuli, particularly those associated with P2Y2R signaling.

### 2.5. Analysis of Gene Expression in Response to UTP in Hepatocytes from CCl4-Induced Fibrotic Mice

These results indicated that treatment with CCl4 sensitized hepatocytes to show an enhanced response to P2Y2R-associated stimuli. Therefore, another aim of this project was to analyze changes in gene expression patterns induced by the UTP-activated P2Y2R, in order to identify the potential mechanisms of purine actions in the liver fibrotic context. To that end, we performed a microarray analysis in which almost 70% of the mouse genome was compared to cDNA obtained from primary cultures of hepatocytes that were isolated from CCl4-treated or control animals and subsequently stimulated with UTP (100 µM) for 24 h (the data of this experiment were deposited in ArrayExpress-EMBL-EBI, accession number: E-MTAB-8302).

#### 2.5.1. Differences in UTP-Dependent Transcriptional Response between Healthy and Fibrotic Hepatocytes

**Figure 5:**
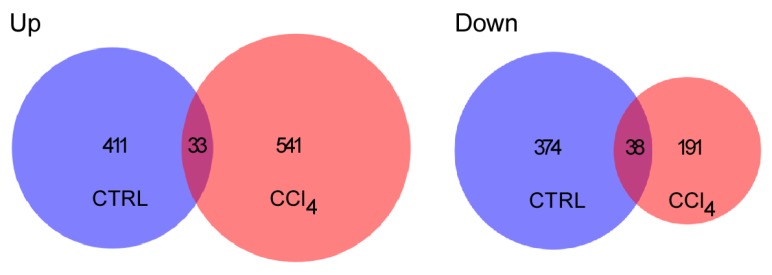
Analysis of gene expression patterns with microarrays of primary cultured hepatocytes stimulated with UTP, isolated from control and fibrotic mice. Venn diagrams representing number of transcripts up- and down-regulated in each condition after UTP stimulation, with the intersection representing the number of transcripts that changed in both settings.

Evident changes were observed in the transcript identity pattern (Figure 5). Marked differences were identified in the number of transcripts that were up-regulated in hepatocytes from the CCl_4_-treated group (541 vs. 411 in the control group; 32% increment, with 33 transcripts in common for both groups); as well as in the down-regulated transcripts, which showed the opposite outcome (191 vs. 374 for CCl_4_-treated and control groups, respectively; a 49% difference, having 38 gene transcripts in common) (Figure 5). Differences were also observed in the transcripts that showed the highest degree of change, related to the z-score within the microarray, in up- and down-regulated transcripts comparing both conditions. Thus, transcripts modified in a control condition after UTP stimulation were related to activities associated with cell maintenance and metabolic pathways, whereas in fibrotic hepatocytes, UTP stimulation promoted a greater response in cell cycle- and DNA damage response-related transcripts (Supplementary Table S1). These results suggested that purinergic stimulation has a differential transcriptional effect on healthy and fibrotic hepatocytes.

#### 2.5.2. Enhanced Proliferative Response in CCl_4_-Treated Hepatocytes

Our results from the MTS experiments indicated that purinergic signaling through the P2Y2R promotes proliferative activity in hepatocytes, therefore an analysis of our microarray results focused on evaluating whether this effect could be appreciated at a transcriptional level. We found several transcripts related with cell cycle and proliferation processes, which were up-regulated in a fibrotic condition after UTP stimulation, such as *Rpa3* and *Rbbp5*, involved in the progression of the cell cycle, as well as *Aatf* and *Zbtb17*, related to avoiding cell death or targeting cell cycle progression inhibitors (Table 1).

#### 2.5.3. Detection of Potential Cellular Mechanisms Regulated by P2Y2R in Fibrotic Hepatocytes

Our aim was to then analyze the microarray results of purinergic stimulation on hepatocytes from CCl_4_-treated animals, using the Gene Ontology tool GeneCodis4, with a focus on Biological Process analysis. Transcripts that showed up-regulated modification were annotated to the Biological pathways of DNA repair (GO:0006281) and cellular response to DNA damage stimulus (GO:0006974) while the down-regulated transcripts were mainly related to negative regulation of transcription by RNA polymerase II (GO:0045944) and negative regulation of cell migration (GO:0030336) (Table 1). Notably, most of the transcripts in the annotated pathways that showed a modification are related to the reparative response that allows for the replication processes (Supplementary Table S2). In addition, one of the results shown by the microarray analysis, in hepatocytes from CCl_4_-treated mice, was the up-regulation of transcripts related to the Hypoxia-Induced Factor (HIF)-1α pathway, such as both the α and β subunit of the HIF-1 complex (z-score = 1.5 and 2.28, respectively), as well as *Vegfa* (z-score= 1.5) which is a known target of the activation of this transcriptional regulator. This was an interesting finding as it was previously reported that the expression of P2Y2R is regulated by the α subunit of the HIF-1 complex [14] and suggests that this pathway could be a mechanism of regulation of the expression of the P2Y2R.

## 3. Discussion

Liver fibrosis is a pathological condition that constitutes the onset of many hepatic diseases with important clinical consequences. There is a growing body of evidence that associates purinergic signaling with the tissue damage response, including the liver [9,15]. Reports show that ATP can be released to the extracellular space by necrotic cells in both acute and chronic damage, promoting specific pathological responses associated with P2 receptors [16,17]. However, the underlying molecular and cellular mechanisms mediated by P2Y receptors in hepatotoxic-induced fibrosis remain to be elucidated. In the present work, we reproduced a well-known animal model of hepatic fibrosis (i.e., hepatotoxic CCl_4_-administration for 4 weeks when fibrosis is still reversible) [10,11] and investigated the expression of P2YR transcripts. Results demonstrated that *P2yr2* and *P2yr6* transcript expression increased considerably in the fibrotic condition, whereas *P2yr13* transcript expression decreased (Figure 2). These observations indicate that the purinergic system is modified by the CCl_4_-induced fibrotic process.

The enhanced expression of the *P2yr6* transcript in CCl_4_-treated mice can correlate with the increase in ERK phosphorylation in response to UDP (Figure 4A), the most potent agonist of this purinergic receptor [18]. In contrast, the response to UDP was undetectable in hepatocytes from control animals, correlating with the low expression of P2Y6R in basal conditions (Figure 2 and Figure 4A). Other reports have described the up-regulation of this receptor in acute and chronic states of inflammation in airway epithelial cells, and although this up-regulation has been associated with an inflammatory response [19,20], several findings have suggested that the role of P2Y6R, similar to that of the P2Y2R, could be mostly proliferative. UDP activation of this receptor can act as a growth factor in rat aortic smooth muscle [21] and also promote a proliferative response in rat theca cells [22,23], thus suggesting that P2Y6R also participates in the damage response in the initial events of the fibrotic process, perhaps by promoting proliferation as an adaptive response.

A decreased expression of the *P2yr13* transcript was also observed. P2Y13R belongs to a different subdivision of P2YRs because it couples to a G-protein with an α_i_ subunit [18]. P2Y13R in the liver has been associated with cholesterol metabolism, favoring the endocytosis of high-density cholesterol-carrying lipoproteins into hepatocytes, as well as their posterior excretion to the biliary duct [24]. It has been documented that elements of the cholesterol metabolism modulate the differentiation of immune cells in innate and adaptive immune systems, the response to inflammatory mediators, and their effects on macrophage migration and function [25]. This physiological process, which could potentially contribute to a successful damage resolution, may be attenuated in a pathological setting by down-regulating elements of this pathway such as P2Y13R. Nonetheless, further experiments are required to assess any changes in the activation and functionality of P2Y13R in the fibrotic condition.

Our results from qPCR, immunofluorescence and Western blot analyses showed an evident increased expression of P2Y2R in the fibrotic condition. Since the signal associated with this receptor was mostly visible in hepatocytes, we performed a set of experiments in the primary cultures of these cells. However, further experiments are required to evaluate specific changes in the expression and function of purinergic signaling in other liver cell types.

To evaluate whether the elevated levels of P2Y2R expression correlated with an increase in cellular responses, ERK phosphorylation and cell viability were analyzed after adding different purinergic agonists, including UTP in primary cultures of hepatocytes, and although this ligand is a selective agonist for P2Y2 and P2Y4Rs, we assumed that the effects are elicited mostly by P2Y2R because the transcript of P2Y4R was undetectable by qPCR. This was further confirmed by a blockade of the UTP-elicited increment in ERK phosphorylation with the P2Y2R-specific antagonist, ARC 118925 (Figure 4B).

We showed that purinergic receptors in isolated mouse hepatocytes in primary cultures are activated by purinergic stimuli (ATP, UTP and UDP). However, when a proliferative response was evaluated in control conditions, there was no significant response to various doses of UTP, except for the smallest dose (10 nM). This unexpected result could be due to hepatocyte heterogeneity. It is well known that hepatocytes are a specialized cell type whose function varies depending on the hepatic lobule zone they occupy [26]. As we observed in our results (Figure 3), P2Y2R in control conditions is not widely distributed throughout the entire tissue; on the contrary, the expression is mainly circumscribed to the pericentral zone. Hence, the effect of purinergic stimulation on hepatocytes in a control condition may comprise a heterogeneous and complex response. On the other hand, in hepatocytes stimulated with UTP (100 µM) from CCl_4_-treated livers, the response of ERK phosphorylation increased more than 5-fold compared to controls. In this condition, we observed a proliferative response with FBS and from the lowest dose of UTP used, which could be due to the wide and homogeneous increase in P2Y2R expression. Our results suggest that hepatocytes from CCl_4_-treated mice are in a sensitized primed state to proliferate in response to purinergic stimuli. In this regard, it has been demonstrated that the reduction in the expression of the ectonucleoside triphosphate diphosphohydrolase 2 (ENTPD2), the enzyme that hydrolases ATP to AMP, facilitated the proliferation of other liver cell types, such as bile duct epithelia [27]. This finding suggests that increasing nucleotide availability in the extracellular space could activate purinergic receptors, including P2Y2R, which could then promote cellular proliferation. P2Y2R is widely expressed in hepatocytes [28]; its activation promotes cellular survival during an inflammatory process associated with acute liver damage [16] and is required for cell cycle progression through the expression of cyclins and other essential elements for efficient hepatocyte proliferation [4]. Furthermore, several findings have associated P2Y2R with proliferative and survival processes, such as partial hepatectomy [29] and hypoxic challenge [30,31], in animal models of liver growth. Supporting our findings, in a model of carcinogenesis-induction by DEN injection, it was recently described that in a P2YR2^-/-^ genetic background, DEN induced a smaller number of tumors and favored an early proliferative response upon toxic administration, indicating that cellular proliferation in response to liver injury, dependent on P2Y2R, is relevant to the liver disease progression [8].

To better understand the potential mechanisms that can be mediated by P2Y2R stimulated by UTP in a fibrotic condition, we used microarrays to evaluate potential variations in transcript expression patterns. First, the analysis showed that UTP promoted distinctive transcriptional patterns in both, control and CCl_4_-treated hepatocytes, revealing notable differences in the role of extracellular purines and P2Y2R activation. Particularly, in the fibrotic condition, transcripts that showed modification after purinergic stimulation were mainly related to proliferation and reparative responses to DNA damage as well as down-regulating the transcription of migration and proliferation inhibitors. These data correlated with our results from MTS experiments, suggesting that during a pro-fibrotic stimulus, purinergic stimulation could suppress inhibitory proliferation signals.

The hepatotoxic CCl_4_, after generating reactive aldehydes, can induce DNA stress and subsequent adduct-formation [32]. It has been demonstrated that elevating the availability of ATP, by attenuating the CD39 ectonucleotidase, promotes a protective response in a DNA-damage situation [33]. Furthermore, the stimulation of DNA damage responses and hepatocyte proliferation has been recently described using another murine model of hepatotoxic administration using DEN [8]. A reparative response to DNA stress for preventing cell death could be an important mechanism by which P2Y2R regulates the regeneration of damaged tissue.

An interesting observation was the down-regulation of transcripts related to the inhibition of the cell migration pathway. It has been described that hepatocytes are able to undergo the process of epithelial-to-mesenchymal transition (EMT), and subsequent cell migration, after the activation of signaling pathways such as the TGF-β pathway [34]. Evidence shows that hepatocytes in primary culture can also sustain EMT after several days in culture, as a result of the loss of organ structure as well as interactions with the other liver cellular types [35]. Moreover, this effect has also been shown after treatment with the hepatotoxic CCl_4_ [36]. Furthermore, it has been described that cellular proliferation, differentiation and migration are processes that play an important role in wound healing responses [37]. Hence, our results could suggest that, after treatment with a hepatotoxic, parenchymal cells show an enhanced phenotypical flexibility and that purinergic stimulation could then synergistically promote cellular migration as an early regenerative response to a stressful context.

On the other hand, among the transcripts that were up-regulated by UTP in hepatocytes from fibrotic livers, both subunits of the HIF-1 complex were detected. It has been widely described that, alongside other enzymatic and metabolic characteristics, oxygen regulates hepatocyte functions throughout the pro-oxidant gradient established between the highly oxygenated periportal zone and the lower oxygenated pericentral zone [26]. Previous evidence has demonstrated that P2Y2R is a direct target of HIF-1α [14], and it is well described that this transcription factor induces the expression of other proliferative and survival genes in hypoxic conditions [38]. Hence, our findings suggest that the HIF-1 complex could be related to the expression of the purinergic receptor in a fibrotic condition to promote a proliferative response as part of the regenerative process after tissue damage. However, further investigations are needed to evaluate whether the increase in both expressions is oxygen-dependent or if there is an independent mechanism where HIF-1α, along with purinergic signaling, can contribute to the onset and development of liver fibrosis.

Overall, in the present study we have demonstrated that, in the CCl_4_-induced fibrotic model, there is a differential expression of P2YRs, where uridine-sensitive receptors increase their expression. In this model, the P2Y2R-specific agonist, UTP, increased ERK phosphorylation and thus proliferative activity, suggesting that the enhanced purinergic signaling through P2Y2R activation in a fibrotic context could be promoting cell proliferation in the hepatic parenchyma during the early stage of hepatic chronic diseases as part of an abnormal regenerative response, and this role could account for the increase in its expression in this condition. Moreover, microarray analysis suggested that P2Y2R activity participates in the positive regulation of DNA-damage and repair responses and in promoting cellular migration by downregulating inhibitors of this process, undoubtedly opening new research directions.

## 4. Materials and Methods

### 4.1. Animals and Induction of Fibrosis with CCl_4_

The induction of fibrosis was done based on previous reports [10,11]. Briefly, male C57BL/6 mice (6–8 weeks old) received CCl_4_ diluted (1:3) in sterile corn oil (0.5 mL of the mix, equivalent to 1.6 g/kg body weight), administered via intraperitoneal (IP) injection three times a week for 4 weeks. Control animals received only corn oil. All the experiments were done in agreement with the Norma Oficial Mexicana de la Secretaría de Agricultura (SAGARPA NOM-062-ZOO-1999), a law that met the guidelines of the Institutional Animal Care and Use Committee Guidebook from the National Institutes of Health-USA; the protocol was approved by the bioethics committee from Instituto de Neurobiología-UNAM (project number 84-A approved in November of 2015).

### 4.2. Histological Analysis

Liver sections were fixed in 10% formalin and embedded in paraffin. Liver slices of 5 µm were stained using standard methods [39]. Hematoxylin and eosin (H&E) staining was used to assess tissue integrity, architecture, cytology and inflammation. Masson´s Trichrome staining was used to outline collagen fibers with blue. The evaluation was done under histopathological criteria [40].

### 4.3. Mitotic Index

Liver samples were fixed in formalin and slices of 5 µm in thickness were obtained. Slices were stained with H&E. Mitotic events were counted in 72 fields from three livers per group (control or CCl_4_) using a 40X objective. The mitotic index was defined as the average of the mitosis found per field [11].

### 4.4. Reverse Transcription and Quantitative Polymerase Chain Reaction (qPCR)

The expression level of *P2ry1, P2ry2, P2ry4, P2ry6, P2ry12, P2ry13* and *P2ry14* transcripts were analyzed by real-time PCR. After perfusion with phosphate buffer (PBS) (in mM: NaCl 136; KCl 2.7; Na_2_HPO_4_ 10; KH_2_PO_4_ 1.8, pH 7.4), a blood-free liver segment was collected, and total RNA was isolated by the guanidine isothiocyanate method [41]. RNA concentration was determined by spectrophotometry in a NanoDrop 1000 (Thermo Scientific, Wilmington, DE, USA) and RNA integrity was corroborated by gel electrophoresis. Reverse transcription was performed from 1 µg of DNAase-treated RNA, with 0.25 µg of oligo dT and 200 U of reverse transcriptase M-MLV (Promega, WI, USA). Reactions of qPCR were made using 3 µL of a 1:5 cDNA dilution with 0.5 µM of oligonucleotides in a commercial qPCR master mix containing SYBR Green I (Roche, Mannheim, Germany), using a LightCycler 2.0 thermal cycler (Roche, Mannheim, Germany).

The oligonucleotides used for the reactions were: *P2yr1forward* TCCAAGAGTGAAGAAATGACTC, *P2yr1reverse* GCTTCTTCTTGACCTGTGTAT, *P2yr2forward* ACCTGGAACCCTGGAATAG, *P2yr2reverse* AGGCGGCATAGGAAGATATAG; *P2yr4forward* CCTGGACTGGACTAAGGAA, *P2yr4reverse* TCAGAGGCAACAGGATGA; *P2yr6forward* TCTGGCACTTCCTCCTAAA, *P2yr6reverse* CTTGAAATCCTCACGGTAGAC; *P2yr12forward* CAGTCTGCAAGTTCCACTAAC, *P2yr12reverse* TGGGTGATCTTGTAGTCTCTG; *P2yr13forward* GAGCTAGTAACTGCCACAAC, *P2yr13reverse CAGGAAGACCACAGTATAGAGA; Cycaforward AGCACTGGGGAGAAAGGATT, Cycareverse AGCCACTCAGTCTTGGCAGT; Sod2forward TGGACAAACCTGAGCCCTAA* and *Sod2reverse GACCCAAAGTCACGCTTGATA.*

The amplification protocol was done as follows: an initial denaturation step of 600 s at 95 °C, followed by 35 cycles at 95 °C for 10 s, 60 °C for 10 s and 72 °C for 12 s. Immediately, a melting analysis was realized with a 0.25 °C/s ramp from 55 to 95 °C. Amplicons were sequenced and analyzed in the Blast platform (NIH, Bethesda, MA) to corroborate their identity. The best fit of housekeeping genes was determined with the Norm Finder software (Department of Molecular Medicine Aarhus University Hospital, Aarhus, Denmark). Transcript abundance was analyzed by the geometric mean of the Ct from two housekeeping genes (*Cyca* and *SOD2)*. Relative quantification was calculated using the Pfaffl model [42].

### 4.5. Immunofluorescence

Liver tissue was fixed in 10% formalin, according to a previously reported protocol [43]. Liver slices of 5 µm were incubated with primary antibody against P2Y2R (Alomone Labs, Jerusalem, Israel) at 1:150 dilution and with secondary antibody donkey anti-Rabbit IgG Cy3 (Thermo Fisher Scientific, Wilmington, DE, USA).

We used ImagePro Plus software (Media cybernetics, MD, USA) to acquire images at 40× magnification and analyze fluorescence. The optical density (OD) was measured in negative (without primary antibody) and positive samples. To obtain the best representative data, we used stratified sampling; three cytoplasmic measures of the same size per hepatocyte were obtained, analyzing 10 hepatocytes from the first line around the central or portal areas of the liver acinus. This procedure was repeated in five images per area for negative and five for positive reaction, both in central and portal areas. In the end, signal quantification involved 150 measurements from negative and positive slides. By subtracting the negative value from the positive signal, the relative levels of fluorescence for central and portal areas were obtained. Graphs represent an average of three mice per group (Control and CCl_4_).

### 4.6. Hepatocyte Isolation

For hepatocyte isolation [44], C57BL/6 mice were anesthetized with a mixture of ketamine/xylazine (1:25 mg/kg of body weight) and placed in a thermostable bed at 37 °C to undergo a laparotomy. After the liver was localized, the hepatic-portal vein was cannulated, and the cava vein was cut to allow solution flow. The liver was then perfused with 50 mL of solution A (NaCl 137 mM, KCl 5 mM, NaH_2_PO_4_ 500 µM, Na_2_HPO_4_ 800 µM, HEPES 9.9 mM, NaHCO_3_ 4.1 mM, EGTA 500 µM, Glucose 5 mM, pH 7.4, at 37 °C) and 50 mL of collagenase (Worthington, Lakewood, NJ, USA) dissolved (1 mg/mL) in solution B (NaCl 137 mM, KCl 5 mM, NaH_2_PO_4_ 500 µM, Na_2_HPO_4_ 800 µM, HEPES 9.9 mM, NaHCO_3_ 4.1 mM, CaCl_2_ 3.8 mM pH 7.4). Then, the liver was dissected in a Petri dish containing 20 mL of William’s E Medium (Sigma Chemical, Saint Louis, MO, USA). Cell suspension was fractioned using Percoll at 10% in Hank’s solution (pH 7.4) to obtain the hepatocyte fraction [45]. Cell viability was analyzed using trypan blue staining. Hepatocytes were cultured for a maximum of 24 h in DMEM-F12 complemented with 10% fetal bovine serum (FBS) and a streptomycin-penicillin mixture at 37°C in an atmosphere of 95% air with 5% CO_2_.

### 4.7. Analysis of the Induction of ERK Phosphorylation and P2Y2R by Western blot

Detection of P2Y2R in mice homogenates as well as the induction of ERK phosphorylation by purinergic agonists in primary cultures of hepatocytes was accomplished by Western blot. For ERK phosphorylation, the induction protocol was as follows. Briefly, hepatocytes were cultured in 12-well plates for 16 h; afterwards, the medium was changed to serum-free medium for 3 h. Then, the experimental treatment (UTP (Sigma Chemical, USA) 100 µM; ARC118925 (Tocris Bioscience UK) 1 µM or both was applied and the hepatocytes were lysed in Laemmli solution (in mM: 125 Tris-HCl, 350 SDS, 200 glycerol, 20 β-mercaptoethanol and 0.03 bromophenol blue); homogenates were collected and boiled for 5 min. Total and phosphorylated ERK were detected through SDS-PAGE according to previously published protocols [22]. In a first blot, p-ERK was detected; afterwards the membrane was stripped with acid glycine solution (200 mM Glycine, 1% tween-20, pH 2.2) and blotted against t-ERK. Primary antibodies were from Cell Signaling Technologies (Danvers, MA, USA), anti-total ERK #4695 and anti-phosphorylated ERK #4370 were used in dilution 1:1000. Secondary antibodies (Invitrogen; Rockford, IL, USA), coupled to horseradish peroxidase (HRP), were used in a 1:5000 dilution. The signal was detected by chemiluminescence and autoradiography. The expression level of P2Y2 receptor in primary cultures of control or CCl_4_-treated hepatocytes was also analyzed by Western blotting; the primary antibody utilized was from Novus Biologicals (Centennial, CO, USA), #NB110-39032 and the protocol used was identical to ERK detection.

### 4.8. Cell Viability Assay

MTS assay (3-(4,5-dimethylthiazol-2-yl)-5-(3-carboxymethoxyphenyl)-2-(4-sulfophenyl)-2H-tetrazolium) was used to measure cell viability, following the manufacturer’s protocol (Promega, USA). Briefly, hepatocytes were seeded into 48-well plates at a cell density of 1 × 10^4^ cells per well in DMEMF-12 with 10% FBS. Cells were then treated with UTP for 24 h. MTS reagent, diluted in serum free medium, was added to each well and incubated for 2 to 4 h until intracellular brown precipitates were visible; then, absorbance was recorded in a 96-well plate and quantified at 490 nm with a Microplate Reader (Bio-Rad, Hercules, CA, USA).

### 4.9. cDNA Microarray Analysis

Microarrays were performed in the Microarray Unit at the Institute of Cellular Physiology (UNAM, CDMX, Mexico). Primary cultures of hepatocytes from control and CCl_4_-treated animals were incubated for 24 h with medium or 100 µM UTP [46]. Briefly, RNA was purified through the Trizol method following the manufacturer’s instructions (Thermo Fisher Scientific, Waltham, MA, USA). Then, cDNA synthesis was achieved with 10 µg of total RNA and using the First-Strand cDNA labeling kit (Thermo Fisher Scientific, Waltham, MA, USA), both dUTP-Alexa555 or dUTP-Alexa647 were incorporated. Fluorescence emission was analyzed at 555 and 650 nm for Alexa555 and Alexa647, respectively. Labeled cDNA was then hybridized against an array of 22,000 transcripts that comprise 70% of the mouse genome; the array contained 65-mer oligo library from Sigma-Genosys (https://www.sigmaaldrich.com/life-science/custom-oligos.html). Array images were acquired and quantified using GenePix 4100A software (OMICtools, RRID:SCR_002250) from Molecular Devices (Sunnyvale, San Jose, CA, USA). Values of mean density of both fluorescent probes and mean background were calculated and microarray data analysis was performed with free genArise software (genArise, RRID:SCR_001346) developed in the Computing Unit of the Institute of Cellular Physiology (UNAM, Mexico) (http://www.ifc.unam.mx/genarise/). GenArise identifies different gene expression patterns by calculating an intensity-dependent Z-score, where z stands for the number of standard deviations a datapoint is from the mean. Considering this criterion, elements with a z-score  >  2 standard deviations would be the transcript genes significantly expressed differentially. To perform the bioinformatics analysis and with the aim of identifying the physiological roles of genes regulated by UTP in both conditions, control and fibrotic, we ran an ontological analysis with the available online software of Gene Ontology, GeneCoDis4 (https://genecodis.genyo.es/) (reviewed from January to February 2020) [47,48,49]. For gene annotation, information was obtained from the database Genecards (https://www.genecards.org/) (reviewed on February 2020). Data were deposited in ArrayExpress-EMBL-EBI, accession number: E-MTAB-8302.

## Figures and Tables

**Table 1:** Gene ontology analysis with the GeneCodis4 tool of signaling pathways and main transcripts modified by purinergic stimulation in CCl_4_-treated hepatocytes.

Up-Regulated Transcripts			Down-Regulated Transcripts		
**GO:0006281 DNA Repair**			**GO:0045944 Negative Regulation of Transcription by RNA Polymerase II**		
Symbol	Z-score	Name	Symbol	Z-score	Name
*Fbxo18*	2.74	F-Box DNA Helicase 1	*Traf7*	−2.86	TNF Receptor Associated Factor 7
*Nthl1*	3.06	Nth Like DNA Glycosylase 1	*Rbl1*	−2.71	RB Transcriptional Corepressor Like 1
*Pold4*	3.81	DNA Polymerase Delta 4, Accessory Subunit	*Cdx4*	−2.65	Caudal Type Homeobox 4
*Rpa3*	5.51	Replication protein A	*Nfib*	−2.37	Nuclear Factor I B
*Eid3*	2.87	EP300 Interacting Inhibitor Of Differentiation 3	*Setdb1*	−2.21	SET Domain Bifurcated Histone Lysine
*Smc3*	3.05	Structural Maintenance Of Chromosomes 3	*Pkig*	−2.16	CAMP-Dependent Protein Kinase Inhibitor Gamma
**GO:0006974 Cellular response to DNA damage stimulus**			**GO:0030336: Negative regulation of cell migration**		
Symbol	Z-score	Name	Symbol	Z-score	Name
*Foxo1*	1.72	Forkhead Box O1	*Nkx2-1*	−2.21	NK2 Homeobox 1
*Aatf*	3.58	Apoptosis Antagonizing Transcription Factor	*Pdgfb*	−2.11	Platelet Derived Growth Factor Subunit B
*Zfp238*	2.27	Zinc Finger And BTB Domain Containing 18	*Dach1*	−2.06	Dachshund Family Transcription Factor 1
*Baz1b*	2.52	Bromodomain Adjacent To Zinc Finger Domain 1B	*Erdr1*	−1.94	Erythroid differentiation regulator
*Uba6*	2.11	Ubiquitin Like Modifier Activating Enzyme 6	*Arid2*	−1.89	AT-Rich Interaction Domain 2
*Rbbp5*	2.27	RB Binding Protein 5	*Rhob*	−1.9	Ras Homolog Family Member B

## Supplementary Materials

Supplementary materials can be found at https://www.mdpi.com/1422-0067/21/7/2305/s1.
