# Supplementary material for: Increased Purinergic Responses Dependent on P2Y2 Receptors in Hepatocytes from CCl4-Treated Fibrotic Mice
Source: Int J Mol Sci. 2020 Mar 26;21(7):2305. doi: 10.3390/ijms21072305 (PMC7177255; doi:10.3390/ijms21072305)
Supplement: Supplementary file 1 [file ijms-21-02305-s001.zip › Supplementary Table 1.pdf]

Supplementary table 1. Main transcripts with modifications after UTP 100 μM stimulation in hepatocytes from control or $CCl_4$ treated conditions.

| **Most up-regulated transcripts** | | | | | |
|---|---|---|---|---|---|
| **VEHICLE** | | | **$CCl_4$** | | |
| Symbol | Z-score | Name | Symbol | Z-score | Name |
| Tfpi2 | 5.59 | Tissue Factor Pathway Inhibitor 2 | U2surp | 5.46 | U2 SnRNP Associated SURP Domain Containing |
| Zmym6 | 5.54 | Zinc Finger MYM-Type Containing 6 | Zbtb17 | 4.53 | Zinc Finger And BTB Domain Containing 17 |
| Asb15 | 5.41 | Ankyrin Repeat And SOCS Box Containing 15 | Ptges3 | 4.19 | Prostaglandin E Synthase 3 |
| Hist1h3g | 5.28 | H3 Clustered Histone 8 | Ralgps2 | 4.00 | Ral GEF With PH Domain And SH3 Binding Motif 2 |
| Grtp1 | 5.25 | Growth Hormone Regulated TBC Protein 1 | Unc119b | 3.83 | Unc-119 Lipid Binding Chaperone B |
| **Most down-regulated transcripts** | | | | | |
| **VEHICLE** | | | **$CCl_4$** | | |
| Symbol | Z-score | Name | Symbol | Z-score | Name |
| Mup5 | -5.07 | Major urinary protein 5 | Six4 | -4.21 | SIX Homeobox 4 |
| C4b | -4.98 | Complement C4B (Chido Blood Group) | Il1rl1 | -3.37 | Interleukin 1 Receptor Like 1 |
| Mup3 | -4.79 | Major urinary protein 3 | Prkci | -3.67 | Protein Kinase C Iota |
| Doc2b | -4.67 | Double C2 Domain Beta | Lilrb4 | -3.65 | Leukocyte Immunoglobulin Like Receptor B4 |
| Mup1 | -4.53 | Major urinary protein 1 | Rbmx | -3.57 | RNA Binding Motif Protein X-Linked |
