# Supplementary material for: Increased Purinergic Responses Dependent on P2Y2 Receptors in Hepatocytes from CCl4-Treated Fibrotic Mice
Source: Int J Mol Sci. 2020 Mar 26;21(7):2305. doi: 10.3390/ijms21072305 (PMC7177255; doi:10.3390/ijms21072305)
Supplement: Supplementary file 1 [file ijms-21-02305-s001.zip › Supplementary Table 2.pdf]

Supplementary table 2. Transcripts regulated by UTP 100 μM by 24h stimulation in hepatocytes from CCl_4-treated mice.

UP-REGULATED TRANSCRIPTS		
**GO:0006281 DNA repair**		
Name	Z-score	Description
Fbxo18	2.74	Plays a key role in response to stalled/damaged replication forks.
Nthl1	3.06	Bifunctional DNA N-glycosylase with associated apurinic/apyrimidinic (AP) lyase function that catalyzes the first step in base excision repair (BER), the primary repair pathway for the repair of oxidative DNA damage.
Pold4	3.81	As a component of the tetrameric DNA polymerase delta complex (Pol-delta4), plays a role in high fidelity genome replication and repair. Induces endothelial cell proliferation.
Rpa3	5.51	As part of the heterotrimeric replication protein A complex (RPA/RP-A), binds and stabilizes single-stranded DNA intermediates that form during DNA replication or upon DNA stress.
Eid3	2.87	Involved in repair of DNA double-strand breaks by homologous recombination.
Smc3	3.05	The nuclear form, known as structural maintenance of chromosomes 3, is a component of the multimeric cohesin complex that holds together sister chromatids during mitosis, enabling proper chromosome segregation.
**GO:0006974 Cellular response to DNA damage stimulus**		
Name	Z-score	Description
Foxo1	1.72	Transcription factor that is the main target of insulin signaling and regulates metabolic homeostasis in response to oxidative stress.
Aatf	3.58	Binds to the pocket region of RB1 and may displace HDAC1 from RB1/E2F complexes, leading to activation of E2F target genes and cell cycle progression.
Zfp238	2.27	Also involved in controlling cell division of progenitor cells and may also play a role in the organization of chromosomes in the nucleus.
Baz1b	2.52	The bromodomain is a structural motif characteristic of proteins involved in chromatin-dependent regulation of transcription.
Uba6	2.11	UBE1L2 is an E1 enzyme that initiates the activation and conjugation of ubiquitin-like proteins. Involved in cellular response to DNA damage stimulus.
Rbbp5	2.27	The encoded protein binds directly to retinoblastoma protein, which regulates cell proliferation.
Shld1	2.33	Shieldin Complex Subunit 1. Component of the shieldin complex, which plays an important role in repair of DNA double-stranded breaks (DSBs).
**DOWN-REGULATED TRANSCRIPTS**		

GO:0045944 Negative regulation of transcription by RNA polymerase II		
Name	Z-score	Description
Traf7	-2.86	TNF Receptor Associated Factor 7. E3 ubiquitin ligase capable of auto-ubiquitination. Induces apoptosis when overexpressed.
Rbl1	-2.71	RB Transcriptional Corepressor Like 1. Directly involved in heterochromatin formation by maintaining overall chromatin structure and, in particular, that of constitutive heterochromatin by stabilizing histone methylation.
Cdx4	-2.65	Caudal Type Homeobox 4. This gene encodes a member of a small subfamily of homeobox containing transcription factors involved in hematopoiesis and negative regulation of transcription.
Nfib	-2.37	Nuclear Factor I B. Transcriptional activator of GFAP, essential for proper brain development. Protein with transcription co-repressor activity.
Setdb1	-2.21	SET Domain Bifurcated Histone Lysine. This gene encodes a histone methyltransferase which regulates histone methylation, gene silencing, and transcriptional repression.
Pkig	-2.16	CAMP-Dependent Protein Kinase Inhibitor Gamma. Extremely potent competitive inhibitor of cAMP-dependent protein kinase activity and is involved in negative regulation of protein import into the nucleus.
**GO:0030336: Negative regulation of cell migration**		
Name	Z-score	Description
Nkx2-1	-2.21	NK2 Homeobox 1. Transcription factor involved in negative regulation of epithelial to mesenchymal transition and cell migration.
Pdgfb	-2.11	Platelet Derived Growth Factor Subunit B. Growth factor that plays an essential role in the regulation of embryonic development and can be involved in negative regulation of vascular smooth muscle cell differentiation.
Dach1	-2.06	Dachshund Family Transcription Factor 1. This gene encodes a chromatin-associated protein that associates with other DNA-binding transcription factors to regulate gene expression and cell fate determination.
Erdr1	-1.94	Erythroid differentiation regulator. Involved in negative regulation of both cell migration and cell population proliferation.
Arid2	-1.89	AT-Rich Interaction Domain 2. This gene encodes a member of the AT-rich interactive domain (ARID)-containing family of DNA-binding proteins. Members of the ARID family have roles in cell lineage gene regulation, cell cycle control, transcriptional regulation and chromatin structure modification.
Rhob	-1.9	Ras Homolog Family Member B. Mediates apoptosis in neoplastically transformed cells after DNA damage. Plays a negative role in tumorigenesis as deletion causes tumor formation.
